# Complementary roles of mitochondrial respiration and ROS signaling on cellular aging and longevity

**DOI:** 10.18632/aging.100485

**Published:** 2012-09-10

**Authors:** Antoni Barrientos

**Affiliations:** Departments of Neurology and of Biochemistry & Molecular Biology, University of Miami Miller School of Medicine, Miami, FL 33136

Although it is widely accepted that mitochondria play fundamental roles in the mechanisms of cellular and organismal aging and lifespan extension, some open questions remain concerning the requirements for aerobic energy production and the effect of the potentially hazardous reactive oxygen species (ROS) byproducts as well as their interplay with nutrition and caloric intake.

We have recently revisited these questions using the *Saccharomyces cerevisiae* chronological life span (CLS) model of aging [1]. CLS models the aging process of post-mitotic cells and measures the capacity of postdiauxic stationary cultures to maintain viability over time [2,3]. For CLS studies, yeast cells are usually aged in media containing 2% glucose. Under these conditions, cells divide exponentially producing energy preferentially by fermentation and respiration is repressed in a glucose concentration-dependent manner. As glucose is being consumed, growth slows down and the diauxic shift occurs which involves a shift from fermentation to respiration, the activation of stress resistance mechanisms and the accumulation of nutrient stores (glycogen and trehalose) to be used later in the stationary phase where the metabolic rate is significantly reduced. Mitochondrial respiration during growth is known to be essential for a strain to achieve a standard wild-type CLS [4,5]. However, our recent data shows that yeast cells have a large reserve respiratory capacity to sustain CLS, as respiration only limits CLS when depleted below a ~40% of wild-type threshold [1]. Strains that respire above the threshold during growth adjust their metabolic rate after the diauxic shift in a manner similar to that of wild-type strains. In contrast, strains that respire below the threshold during growth have extremely poor respiratory capacity in the stationary phase and very short CLS. Further, respiratory defects are less detrimental when produced only in the stationary phase, once yeast have accumulated nutrient stores during growth and undergone their metabolic remodeling during the diauxic shift.

An essential question is why strains respiring below the threshold have a short CLS. First, aerobic energy production is required for the efficient function of anti stress systems, most needed during the diauxic shift and stationary phase. Furthermore, cells respiring below the threshold have altered metabolism of stored nutrients. Respiratory-deficient cells accumulate normal amounts of glycogen during growth, but they synthesize trehalose inefficiently. Both of these reserve carbohydrates are quickly consumed when the cells reach the stationary phase because, in the absence of respiration, yeast must rely on fermentation, which generates a 14-fold lower energetic yield. Glycogen and trehalose are virtually exhausted in respiratory-deficient cells by the end of their CLS, thus suggesting that starvation is an important factor limiting their survival. In support of this conclusion, trehalose supplementation of the growth media enhances the stress-resistance capacity of respiratory null strains and significantly extends their CLS.

Another way in which mitochondrial respiration controls aging and longevity is through generation of ROS. Although mitochondrial ROS are known to limit the long-term survival of yeast cells during CLS and can have a detrimental effect during aging in higher eukaryotes, they can also act as signaling molecules with hormetic effects on longevity [6-8]. For example, tor1Δ strains deficient in the nutrient-responsive TORC1 signaling have increased coupled respiration, and also enhanced ROS production during exponential growth that provides an adaptive signal that contributes to increase cell protection systems and extend CLS [6]. A comparison of yeast strains with different genetic backgrounds has suggested a correlation between respiratory capacity, mitochondrial ROS generation and CLS [1]. Strains with high respiration and ROS production have longer CLS which is curtailed by increased mitochondrial superoxide dismutase SOD2 levels while strains with low respiration and ROS production have shorter CLS and can significantly benefit from additional exogenous ROS or tor1Δ-induced endogenous ROS to significantly extend their CLS [1,6]. Also caloric restriction (CR) extends the lifespan in yeast through a mechanism that involves, in part, inhibition of nutrient-responsive kinases such as TOR and RAS2 [9], general enhancement of stress-resistance mechanisms, increased respiration during growth and metabolic remodeling involving a dramatic reduction in their metabolic rate in the stationary phase that allow the cells to consume their stored nutrients at a slow rate. However, cells must respire during growth at least above a ~40% threshold to benefit from either CR- or tor1Δ-induced CLS extension. Also important, increased respiration during growth (i.e. by hyperactivating mitochondrial biogenesis) is not sufficient to extend CLS in strains that have maximized the CLS extension achievable by ROS signaling if it is not accompanied by an enhancement of cell protection systems that promote survival in the stationary phase as for tor1Δ and CR-treated cells.

**Figure 1:**
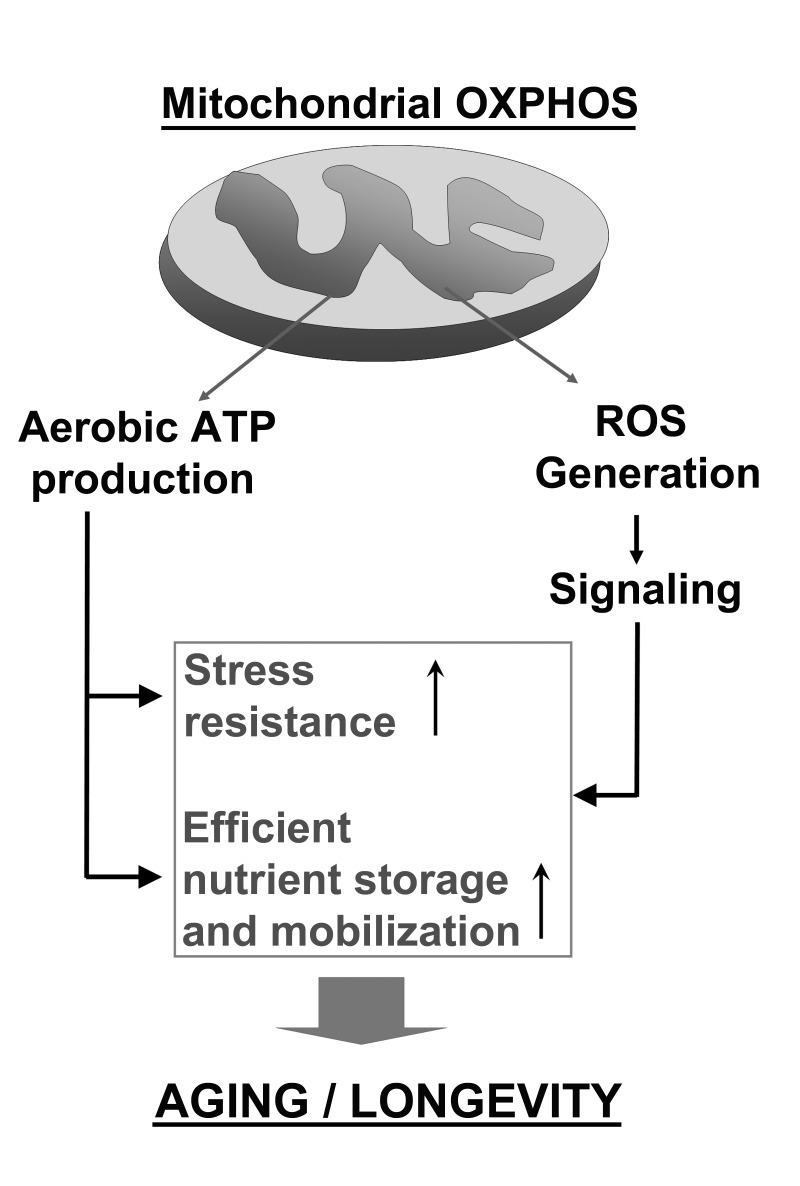
Complementary roles of mitochondrial energy production though respiration and oxidative phosphorylation (OXPHOS) and mitochondrial ROS signaling in promoting cell survival and regulating aging and longevity.

In conclusion, we have proposed a model (Figure 1) in which ROS signaling and respiratory thresholds contribute to achieve increased stress resistance, efficient use of energy stores, and likely other beneficial effects in stationary phase, which extends CLS. In multicellular organisms, respiratory thresholds to support cellular life probably vary for every tissue, which in turn likely changes the importance of ROS signaling in a tissue-specific manner. Further studies on the dynamic changes of these mitochondrial parameters in response to environmental cues are expected to provide important information concerning cellular, tissular and organismal aging.
